# Influence of the physical effort of reminder-setting on strategic offloading of delayed intentions

**DOI:** 10.1177/17470218231199977

**Published:** 2023-09-23

**Authors:** Gavin Chiu, Sam J Gilbert

**Affiliations:** Institute of Cognitive Neuroscience, University College London, London, WC1N 3AZ, UK

**Keywords:** Memory, effort, metacognition, offloading, reminder

## Abstract

Intention offloading involves using external reminders such as diaries, to-do lists, and digital alerts to help us remember delayed intentions. Recent studies have provided evidence for various cognitive and metacognitive factors that guide intention offloading, but little research has investigated the physical cost of reminder-setting itself. Here, we present two pre-registered experiments investigating how the cost of physical effort associated with reminder-setting influences strategic intention offloading under different levels of memory load. At all memory loads, reminder-setting was reduced when it was more effortful. The ability to set reminders allowed participants to compensate for the influence of memory load on accuracy in the low-effort condition; this effect was attenuated in the high-effort condition. In addition, there was evidence that participants with less confidence in their memory abilities were more likely to set reminders. Contrary to prediction, physical effort had the greatest effect on reminder-setting at intermediate memory loads. We speculate that the physical costs of reminder-setting might have the greatest impact when participants are uncertain about their strategy choice. These results demonstrate the importance of physical effort as one of the factors relevant to cost-benefit decision-making about cognitive offloading strategies.

Human cognition often relies on the body and external environment to achieve competent behaviour. We point with our fingers to indicate locations, tilt our heads when facing rotated objects (Risko et al., 2014), and arrange objects so that we remember where they were put (Berry et al., 2019). These are examples of *cognitive offloading*, a term defined by Risko and Gilbert (2016) as physical action used to alter the information processing requirements of a task to reduce cognitive demand.

A subset of cognitive offloading concerns the way we remember and execute intentions. Typically, intentions cannot be fulfilled immediately, and there exists an interval between the formation of an intention in our mind and its execution, which is why we often need cues from the environment to trigger the retrieval of stored intentions (Crystal & Wilson, 2015). The process of creating an artificial cue in the external environment to trigger a delayed intention is known as *intention offloading* (Gilbert et al., 2023). This artificial cue can come in different forms. It can be an organisation of the environment; for example, when intending to post a letter the next time we leave the house, we might place the letter close to the door. It can be created on other individuals, e.g., asking your colleague to remind you of a meeting tomorrow. But most commonly, we offload intentions by using external props and tools as reminders, such as to-do lists, schedule books, sticky notes, and digital reminders. As a result of offloading, we store a relevant cue in our extended physical and social environments to minimise the chance of memory failure (Gilbert et al., 2023).

Intention offloading relies on a process of cost–benefit decision-making (Gilbert, 2023; Gilbert et al., 2020). Although reminders are undoubtedly helpful, it is not possible to set a reminder for every intention we have. Because of the time and effort it takes to offload an intention, we often have to decide when to use reminders and what to use them for. Consistent with this, people are more likely to set reminders for intentions when they are associated with a higher financial reward (Dupont et al., 2023).

Recent research has begun to investigate other factors that influence this evaluation. These include task difficulty (including both memory load and the presence of interruption in the ongoing task; Gilbert, 2015a), strategy perseveration (Scarampi & Gilbert, 2021), and cognitive effort avoidance (Sachdeva & Gilbert, 2020). Cognitive offloading strategies are influenced by metacognitive judgements (i.e. people tend to set reminders when they *think* that they will otherwise forget, not necessarily when they will *actually* forget; Boldt & Gilbert, 2019, 2022; Gilbert, 2015b; Gilbert et al., 2020) and are associated with age-related change (Bulley et al., 2020; Redshaw et al., 2018; Scarampi & Gilbert, 2021; Tsai et al., 2023). For a review of these findings, see Gilbert et al. (2023).

Gilbert (2023) recently proposed that the process of cognitive offloading can be seen as a form of value-based decision-making, balancing the benefits of offloading (e.g. the increased likelihood of remembering and the ability to reallocate memory capacity to other information) against its costs (e.g. the time and physical activity required to set up a reminder). However, these costs have not yet been systematically explored in an experimental setting. To investigate this, the present study adapted a previous task used to investigate reminder-setting strategies (Gilbert et al., 2020) and manipulated both the number of intentions (i.e. memory load) and the physical effort it took to set a reminder. Our main interest was whether the physical effort of reminder-setting affected offloading strategies, and if so whether this effect varied between different memory loads. Six pre-registered hypotheses (https://osf.io/qky7j/) corresponding to three groups of analyses were proposed in this study:

*Analysis 1: Task Performance*
Hypothesis 1: Accuracy will decrease at higher memory loads.
Hypothesis 2: Accuracy will increase when participants are allowed to set reminders.
*Analysis 2: Offloading Behaviour*
Hypothesis 3: When given a free choice, participants will set more reminders at higher memory loads.
Hypothesis 4: Participants will set fewer reminders when this requires more physical effort.
*Analysis 3: Metacognitive Judgement*
Hypothesis 5: Participants will be underconfident in their predicted accuracy relative to their actual performance. This is based on findings from previous studies using related paradigms (Engeler & Gilbert, 2020; Gilbert et al., 2020; Kirk et al., 2021).
Hypothesis 6: Participants with lower confidence in their unaided memory ability will be more likely to set reminders (Boldt & Gilbert, 2019; Gilbert, 2015b; Kirk et al., 2021).

In addition to these main hypotheses, we conducted exploratory analyses to investigate whether the influence of physical effort differs between memory loads. We predicted that physical effort might suppress reminder-setting more strongly at lower memory loads (when reminders are less necessary) than at higher memory loads. We also tested whether task performance in unaided trials correlated with offloading behaviour when reminders were allowed. This correlation would suggest that people with weaker objective memory abilities are more inclined to use reminders to support their memory.

## Experiment 1

### Methods

Before commencing data collection, all experimental hypotheses, designs, procedures, and analyses were pre-registered at https://osf.io/qky7j/. All deviations from this pre-registered plan are clearly labelled.

#### Participants

A total of 52 participants were recruited online via Prolific (https://www.prolific.co).We excluded outlying participants whose mean target accuracy, averaged across all memory load and offloading conditions, exceeded 2.5 Median Absolute Deviation units from the full sample (Chen et al., 2021). Three participants were excluded based on this criterion and were replaced with another three participants. The final sample consisted of 52 participants (28 male, 24 female; mean age: 25 years; range: 19–41  years).

The above sample size was estimated by power calculation performed using G*Power 3.1. We wanted to power our study to detect the effect of changing physical effort on individual offloading behaviour. As no previous literature, to our knowledge, has investigated this effect, we predicted our effect size based on a similar study conducted by Sachdeva and Gilbert (2020). Their study used a similar experimental paradigm to demonstrate that providing financial reward as an incentive to use internal memory significantly reduced reminder-setting with an effect size of *d* = 0.51. We proposed that increasing physical effort associated with reminder-setting and providing financial rewards could have a main effect of comparable magnitude in reducing reminder-setting. We, therefore, decided to collect a sample so that it would have sufficient power to detect an effect of this size in a within-subjects design (*dz* = 0.51), with a two-tailed test, alpha set to 0.05 and a power of 95%. The projected sample size required for this study was *n* = 52.

The entire experiment was advertised to last approximately 25 minutes. Each participant was paid £3 after completing the experiment. To incentivise task performance, participants were also told that they would receive an additional £1 bonus if they performed in the top 50% of the entire sample. All participants provided informed consent before participation, and the study was approved by the UCL (University College London) Research Ethics Committee (1584/003).

#### Intention offloading task

The task used in this study was programmed in Java using Google Web Toolkit version 2.7.0 (http://www.gwtproject.org) implemented in the Eclipse development environment version 4.14.0 (https://www.eclipse.org). To view the experiment, visit http://samgilbert.net/demos/GC1. The code that runs the entire experiment can be found by visiting https://github.com/MetaOffloading/GC1.

**Figure 1.**
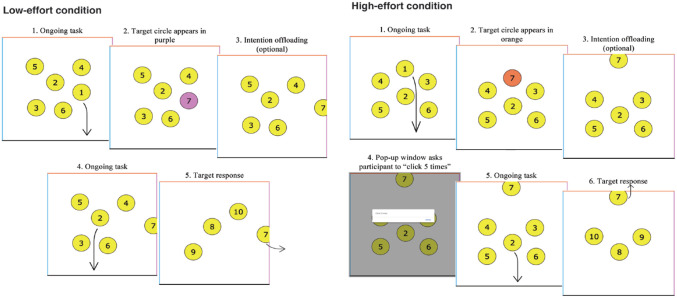
Schematic representation of the intention offloading task used in the current study (low-effort and high-effort conditions).

See Figure 1 for a schematic illustration of the task. Participants performed a web-based intention offloading task individually on their computers or tablets but were prohibited from using smartphones. The task was modified from the optimal reminders paradigm developed by Gilbert et al. (2020). At the beginning of each trial, participants viewed six numbered yellow circles randomly positioned inside a square on the screen. They were instructed to use their mouse (or their finger if performed on a tablet) to drag the circles to the bottom of the square in ascending numerical order (1, 2, 3, etc.). Each time a circle was dragged to the bottom, it would disappear, and a new circle following the sequence would replace the dragged circle in its original location, for example, after circle 1 was dragged to the bottom, circle 7 would appear in circle 1’s original position.

During the ongoing task of circle dragging, participants were provided with delayed intentions. Circles that appeared from circle 7 onwards were special circles. These special circles would appear on the screen in different colours corresponding to a specific side of the square (left: blue, right: purple, top: orange). The circles would fade back to yellow after 2 s. As participants were dragging regular circles 1–6, they were tasked to remember the colours of these special circles and drag them to their respective sides upon reaching that number in the sequence. For example, imagine a situation where circle 1 was dragged to the bottom, and circle 7 appeared in purple. After 2 s, circle 7 would fade back to yellow, and the participant would continue dragging circles 2–6, while remembering the special colour associated with circle 7. When the participant finally reached circle 7 in the sequence, they would drag it to the right instead of the bottom because the right side was associated with purple. Hence, a delayed intention was formed when the participant first saw circle 7 in purple. In each trial of the experiment, there were two, four, or six special circles corresponding to three levels of memory load. As all special circles appeared from circle 7 onwards, it meant that each trial contained eight, ten, or twelve circles in total.

Participants could rely entirely on their unaided memory to perform the task. Alternatively, they could offload their intentions by setting reminders. Participants were taught a simple offloading strategy that was integrated into the circle-dragging task. As soon as a special circle appeared on screen, they could immediately drag it close to its target side. This new location would become a perceptual trigger to remind the participant where the circle should go when that number was reached. For instance, when the participant first saw circle 7 in purple, they could drag it towards the right before continuing with circles 2–6. Once circle 7 was reached, the participant would know by the circle’s location that it should go to the right. By employing this strategy, delayed intentions were offloaded to the external environment as participants no longer had to remember or mentally rehearse the specific colours of the special circles.

#### Counterbalancing and effort manipulation

The experiment involved manipulations of two key factors—memory load and offloading conditions. Memory load, as mentioned above, was controlled by changing the number of special circles (two, four, or six) presented to the participant. Before each trial, participants were told how many special circles they would get so that they could prepare and formulate a memory strategy. Three offloading conditions were presented in total—no reminder (trials where participants were forced to use internal memory), low-effort reminders (trials where reminders were optional and required low physical effort), and high-effort reminders (trials where reminders were optional and required high physical effort).

Each participant performed three blocks of trials in the experiment, corresponding to the three offloading conditions. Each block contained nine trials of the same circle-dragging task. Within the nine trials, three contained two special circles, three contained four special circles, and another three contained six special circles. Trials in each block were presented to the participants in random order. The experiment was randomised in both “reminder condition” and “effort condition” for counterbalancing purposes. Randomising the “reminder condition” meant that some participants were assigned to perform the no-reminder block first, then moved on to the two reminder blocks (“no-reminder first” condition); whereas others performed the two reminder blocks before the no-reminder block (“reminders first” condition). Randomising the “effort condition” meant that within the two reminder blocks, some participants were first taught to use low-effort reminders, then switched to high-effort reminders (“low-effort first” condition); whereas the rest would do the opposite (“high-effort first” condition). The two reminder blocks were always placed adjacent to one another, to increase the salience of the difference in physical effort.

In terms of how physical effort was manipulated, we incorporated a physically demanding clicking task after each reminder was set in the high-effort block (see Figure 1 above). Upon moving a special circle close to its target side, a window would pop up on screen and the participant had to click and close the window five times before they could proceed to move the next circle. A real-world analogue of this high-effort reminder might be a smartphone reminder app with a poorly designed user interface that requires users to perform multiple unnecessary clicks to set a reminder. On the contrary, in the low-effort block, participants would not engage in any clicking task after each reminder, and they could freely proceed to the next circle after setting a reminder.

#### Procedures

Participants began by performing four practice trials in the following order: no special circle, two special circles, four special circles, six special circles. All circles except the next one in sequence were fixed on screen, meaning that participants were forced to rely on their own memory. Afterwards, they were asked to evaluate how accurately they thought they could perform the task at each memory load (i.e. self-predicted accuracy). As this evaluation took place before any offloading strategy was taught, it reflected participants’ metacognitive confidence in their internal memory.

Next, the offloading strategy was taught. Participants in the “low-effort first” condition were first taught the low-effort strategy without the additional clicking task, whereas those in the “high-effort first” condition were taught the strategy with the clicking task. All participants performed one practice trial with four special circles using their respective taught strategies.

Participants in the “no-reminder first” condition then proceeded to perform a block of nine trials of the circle-dragging task. In this block, they were instructed not to use any offloading strategy—the system also disabled reminder-setting by fixing all circles that were not next in the sequence. Participants were thus required to use internal memory to remember as many special circles as possible. After that, they moved on to the next part of the experiment, where reminders were allowed.

For those in the “low-effort first” condition, they performed another block of nine trials, this time with the option to freely choose between setting reminders with the taught low-effort strategy or using internal memory. After completion, they were informed that reminder-setting would now become more effortful and were introduced to a modified strategy with the additional clicking task. They then performed another practice with four special circles and advanced to the final block of the experiment, where they repeated the previous block but with high-effort reminders.

For those in the “high-effort first” condition, procedures were essentially the same, except participants first used the high-effort strategy, then were told reminder-setting would become less effortful and subsequently switched to the low-effort strategy. Similarly, participants in the “reminders first” condition also went through the same procedures but with one difference: they performed the two reminder blocks first before attempting the no-reminder block. At the end of the experiment, all participants were thanked for their time and paid.

#### Data analysis

All data were analysed using R version 4.1.2 and RStudio version 2021.09.0. The data and code to reproduce all analyses can be found at https://osf.io/qky7j/. Data were analysed by repeated measures analysis of variance (ANOVA), with follow-up pairwise comparisons using paired samples *t*-tests. Results were two-tailed with a significance threshold of *p* < .05. Degrees of freedom in the repeated measures ANOVAs were adjusted by a Greenhouse-Geisser correction where the assumption of sphericity was not met. All analyses were conducted as described in our pre-registered plan (https://osf.io/qky7j/). In addition, several exploratory analyses were performed, which were not included in the plan. They will be described in the Results section below and clearly labelled as not being pre-registered. Independent measures were three levels of memory load (two, four, and six special circles) and three levels of offloading conditions (no reminder, low-effort reminders, and high-effort reminders) manipulated in a within-subject manner. Key dependent measures were:

Target accuracy: the proportion of special circles correctly dragged to their target sides of matching colour, instead of to the bottom of the square (adopted from Gilbert, 2015a).

Externalising proportion: the proportion of special circles for which the participant sets an external reminder. In common with previous studies (e.g., Gilbert, 2015a), this was measured by calculating the likelihood of dragging a target circle when it was not the next in the numerical sequence and then subtracting the equivalent likelihood from nontarget circles. This corrects for any general tendency to accidentally click the wrong circle.

Metacognitive evaluation: a self-predictive measure with a range of 0% to 100% showing how confident participants thought they could accurately perform the task without external reminders (adopted from Gilbert et al., 2020).

### Results

#### Target accuracy

**Figure 2.**
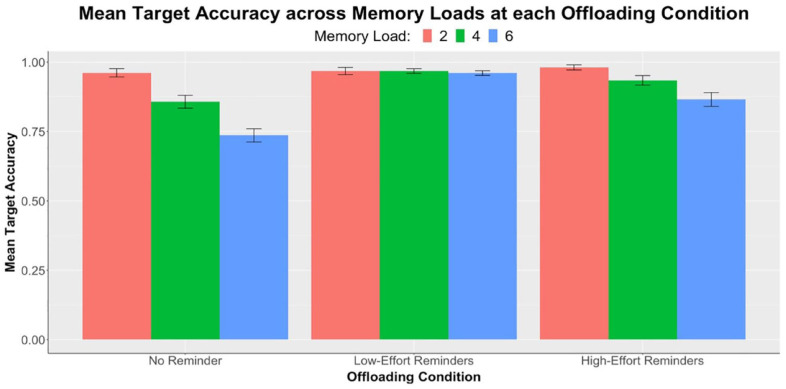
Experiment 1: Results of mean target accuracies (*n* = 52) across three levels of memory load, separately at each offloading condition. Error bars represent standard errors corrected for the within-subjects design using the method of Morey (2008).

A summary of the target accuracy data is illustrated in Figure 2. We first conducted a 3 × 3 repeated measures ANOVA with the dependent variable of target accuracy and factors of memory load (two, four, and six) and offloading condition (no-reminder, low-effort, and high-effort). There was a significant main effect of memory load, *F*(1.81, 92.50) = 47.13, *p* < .001, η_p_^2^ = .48. We followed up by performing one-way repeated measures ANOVAs and pairwise comparisons between memory loads at each offloading condition. Results are displayed in Table 1. In line with our prediction, significant pairwise differences across all memory loads were observed in the no-reminder condition, showing that participants performed increasingly poorly as the memory load increased. The same effect was also observed in participants using high-effort reminders; on the contrary, accuracy differences across memory loads in the low-effort condition remained nonsignificant.

**Table 1.** Experiment 1: One-way ANOVAs (*F*-values, significance levels, and partial eta-square) and pairwise comparisons (*t*-values, effect sizes, and significance levels) of mean target accuracy between memory loads at each offloading condition. Refer to Figure 2 for mean differences.

	Offloading condition					
	No reminder		Low-effort reminders		High-effort reminders	
One-way repeated measures ANOVA	*F*(1.88, 96.43) = 43.87***, η_p_^2^ = .46		*F*(1.79, 91.11) = .21, η_p_^2^ = .004		*F*(1.48, 75.67) = 11.64***, η_p_^2^ = .186	
Pairwise comparisons between memory loads	*t*(51)	*d_z_*	*t*(51)	*d_z_*	*t*(51)	*d_z_*
Memory loads						
2 vs. 4	4.91***	0.68	<.01	<.01	2.83**	.32
4 vs. 6	4.97***	0.69	.70	.10	2.35*	.33
2 vs. 6	8.54***	1.18	.53	.07	4.67***	.65

ANOVA: Analysis of variance.

† *p* < .1. *p < .05. **p < .01. ***p < .001.

In the same 3 × 3 ANOVA, a significant main effect of offloading condition was also found, *F*(1.73, 88.29) = 38.90, *p* < .001, η_p_^2^ = .43. We performed pairwise comparisons between the three offloading conditions at each memory load, as shown in Table 2. We predicted that reminder-allowed accuracy, in any effort condition, would be significantly higher than no-reminder accuracy at all memory loads. Results partially supported our hypothesis, with significant differences observed only in memory loads 4 and 6 but not in 2. Interestingly, at memory load 6, target accuracy in the high-effort condition was significantly lower than that in the low-effort condition. This was evidence that the increase in physical effort associated with setting a reminder negatively influenced participants’ target accuracy at high memory loads.

**Table 2.** Experiment 1: Pairwise comparisons (*t*-values, effect sizes, and significance levels) of mean target accuracy between offloading conditions at each memory load. Refer to Figure 2 for mean differences.

Pairwise comparisons between offloading conditions	Memory load					
	2		4		6	
	*t*(51)	*d_z_*	*t*(51)	*d_z_*	*t*(51)	*d_z_*
Offloading conditions						
No reminder vs. low-effort reminders	.39	.05	5.28***	.73	9.16***	1.27
No reminder vs. high-effort reminders	1.10	.15	3.22**	.45	4.33***	0.60
Low-effort reminders vs. high-effort reminders	0.81	.11	1.93^†^	.27	3.82***	0.53

† *p* < .1. *p < .05. **p < .01. ***p < .001.

The memory load x offloading condition interaction in this 3 × 3 ANOVA was significant, *F*(3.09, 157.38) = 13.19, *p* < .001, η_p_^2^ = .21. We conducted additional 2 × 3 repeated measures ANOVAs as the follow-up to examine interactions for each pairwise comparison between two of the three offloading conditions. Interactions were consistently significant across each pair: no-reminder and low-effort: *F*(1.84, 93.96) = 36.70, *p* < .001, η_p_^2^ = .42; no-reminder and high-effort: *F*(1.74, 88.64) = 5.03, *p* = .011, η_p_^2^ = .09; low-effort and high-effort: *F*(1.91, 97.40) = 6.87, *p* = .002, η_p_^2^ = .12. These interactions demonstrated that the effect of memory load on target accuracy was greater in the no-reminder condition than the other two reminder-allowed conditions. The effect was also greater in the high-effort condition compared with the low-effort condition.

#### Externalising proportion

**Figure 3.**
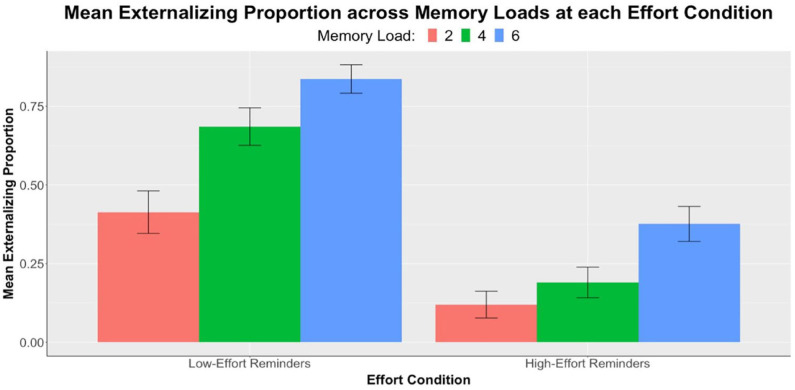
Experiment 1: Results of mean externalising proportions (*n* = 52) across three levels of memory load, separately at each effort condition. Error bars represent standard errors corrected for the within-subjects design using the method of Morey (2008).

Next, we investigated participants’ offloading behaviour across memory loads and between the two effort conditions. Figure 3 below shows a summary of our externalising proportion data. We used a two-factor 2 × 3 repeated measures ANOVA with the dependent variable of externalising proportion and factors of memory load (two, four, and six) and effort conditions (low-effort, high-effort). As expected, main effect of memory load was significant, *F*(1.88, 95.92) = 38.83, *p* < .001, η_p_^2^ = .43. Follow-up pairwise comparisons confirmed significant differences in externalising proportion across memory loads at each effort condition, with only one exception in the comparison of load 2 vs. 4 in the high-effort condition. Details are shown in Table 3 above. This result provided evidence that people generally set more reminders when the memory load was higher.

**Table 3.** Experiment 1: Pairwise comparisons (*t*-values, effect sizes, and significance levels) of mean externalising proportion between memory loads at each effort condition. Refer to Figure 3 for mean differences.

Pairwise comparisons between memory loads	Effort condition			
	Low-effort reminders		High-effort reminders	
	*t*(51)	*d_z_*	*t*(51)	*d_z_*
Memory loads				
2 vs. 4	5.57***	0.77	1.72^†^	.24
4 vs. 6	3.30**	0.46	4.29***	.60
2 vs. 6	7.44***	1.03	5.33***	.74

† *p* < .1. *p < .05. **p < .01. ***p < .001.

We then turned to the key analysis of our study: the effect of increasing physical effort on offloading behaviour. A significant main effect of effort condition was found, *F*(1, 51) = 90.81, *p* < .001, η_p_^2^ = .64. Consistent with this, follow-up analyses with paired samples *t*-tests demonstrated significant differences in externalising proportion between low-effort and high-effort conditions at all memory loads. The differences are shown in Table 4. Results, therefore, supported our hypothesis: increasing the physical effort of reminder-setting significantly reduced offloading behaviour at all memory loads.

**Table 4.** Experiment 1: Comparison of externalising proportion between effort conditions at each memory load (mean differences, *t*-values, effect sizes, and significance levels).

Paired samples *t-*tests comparing low-effort vs. high-effort reminders at each load			
	Mean difference	*t*(51)	*d_z_*
Memory loads			
2	.29	5.09***	0.71
4	.50	9.33***	1.29
6	.46	8.88***	1.23

† *p* < .1. *p < .05. **p < .01. ***p < .001.

As part of our exploratory analyses, we further examined the interaction between memory load and effort condition to study if people were more selective in offloading when effort increased. The interaction was significant, *F*(1.85, 94.27) = 7.45, *p* = .001, η_p_^2^ = .13, suggesting that the reduction in externalising proportion between low- and high-effort conditions was not constant across memory loads. However, contrary to what we predicted, data did not show a larger reduction in offloading at memory load 2 compared with load 6. Instead, an opposite effect was observed (see Table 4). Our results demonstrated that increasing physical effort had a greater effect on reducing offloading behaviour at higher memory loads. Hence, our exploratory hypothesis concerning effort-induced selectivity was not supported by this interaction analysis.

#### Metacognitive evaluation

**Figure 4.**
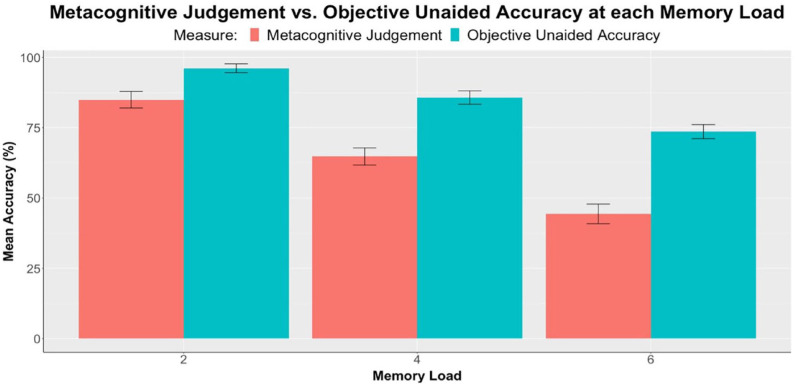
Experiment 1: Comparison between metacognitive judgements and objective unaided accuracy (*n* = 52), separately at each memory load. Error bars represent standard errors corrected for the within-subjects design using the method of Morey (2008).

Figure 4 shows participants’ mean metacognitive judgements and objective unaided accuracies, that is, mean performance in the no-reminder condition, at each memory load. The difference between these two measures indicated metacognitive bias, that is, the extent of underconfidence.

A 2 × 3 repeated measures ANOVA was conducted with a dependent variable of accuracy and factors of memory load (two, four, and six) and measure (metacognitive judgement and objective unaided accuracy). Results demonstrated a significant effect of memory load, *F*(1.45, 73.80) = 98.60, *p* < .001, η_p_^2^ = .66. More importantly, the main effect of measure was also significant, *F*(1, 51) = 118.78, *p* < .001, η_p_^2^ = .70, and follow-up paired samples *t*-tests comparing the two measures at each load all produced significant differences (see Table 5). Self-predicted accuracies, that is, metacognitive judgement, were significantly lower than objective unaided accuracies at all memory loads. It was thus evident that participants were generally underconfident in their internal memory abilities. Besides, there was a significant interaction between memory load and measure, *F*(1.56, 79.71) = 7.81, *p* = .002, η_p_^2^ = .13, implying that the extent of underconfidence increased as memory load increased.

**Table 5.** Experiment 1: Comparison between metacognitive judgements and objective unaided accuracy at each memory load (*t*-values, effect sizes, and significance levels). Refer to Figure 4 for mean differences.

Paired samples *t-*tests comparing metacognitive judgements vs. objective unaided accuracy at each load		
	*t*(51)	*d_z_*
Memory loads		
2	3.78***	0.52
4	6.62***	0.92
6	8.22***	1.14

† *p* < .1. *p < .05. **p < .01. ***p < .001.

Our final hypothesis concerning the correlation between confidence and offloading was not supported by our results. Correlation analyses between mean metacognitive judgements/bias and mean externalising proportion, averaged across all memory load and effort conditions, were first performed. Both correlations were nonsignificant despite being negative, metacognitive judgement: *r*(50) = −.16, *p* = .24; metacognitive bias: *r*(50) = −.09, *p* = .52. We then followed up by running the same correlations separately at each memory load, averaged across effort conditions. Most of the correlations under these different conditions were also found to be nonsignificant (see Tables 1 and 2 in the online Supplementary Material for details of correlations).

#### Other analyses

In our final exploratory hypothesis, we wanted to examine if participants who scored lower in no-reminder trials would set more reminders when they were allowed. A negative correlation between these two variables was therefore predicted. Analysis between mean unaided accuracy and mean externalising proportion averaged across all memory load and effort conditions, did reveal a significant negative correlation, *r*(50) = −.34, *p* = .01. However when we performed the same analysis separately at each memory load and effort condition, correlations became mostly nonsignificant (see Table 3 in the online Supplementary Material). Hence, from the results of this study, there seemed to be a negative relationship between internal memory ability and offloading behaviour only when collapsing across all memory load and effort conditions, but this relationship was weak and nonsignificant when looking individually at each condition. We note that the original power calculation was based on the effect of physical effort on reminder-setting, not these exploratory analyses; hence, the study may have been underpowered to detect correlations.

### Discussion

This experiment demonstrated the clear influence of physical effort on intention offloading. We had multiple predictions prior to the investigation, most of which were supported by our results. First, in situations where reminders were not allowed or were effortful, task performance was found to be negatively associated with memory load. The drop in accuracy was smaller in the high-effort condition compared with the no-reminder condition (see effect sizes in Table 1) but was nevertheless significant. These findings were in line with existing literature showing a similar negative effect of memory load on target accuracy (Gilbert, 2015a; Redshaw et al., 2018; Scarampi & Gilbert, 2021). Two additional improvements were made in our experiment with regard to methodology: we manipulated three levels of memory load (two, four, and six targets) instead of two levels in previous studies (one target vs. three targets) (Gilbert, 2015a; Redshaw et al., 2018; Scarampi & Gilbert, 2021) to demonstrate a decreasing trend of accuracy as memory load increased; we also embedded intentions *during* rather than *before* the circle-dragging task (Gilbert, 2015a; Redshaw et al., 2018; Scarampi & Gilbert, 2021) to create greater real-world resemblance where intentions are usually formed and held as we engage in ongoing tasks at hand.

Notably, at the low-effort condition, there was no statistically significant change in accuracy across memory loads (see Table 1, Figure 2). This suggests that when physical effort was low, the availability of reminders enabled compensation for the increase in task difficulty. Participants were aware of the benefits brought by setting reminders and, therefore, managed to achieve similar levels of accuracy by strategically using more reminders at higher memory loads. This observation was further substantiated by the increase in externalising proportion as memory load increased (see Figure 3). By contrast, the decrease in accuracy across loads in the high-effort condition suggests that participants failed to fully adjust for task difficulty when reminders became more effortful. It was also found that at load 6, accuracy in the high-effort condition was significantly lower than that in the low-effort condition. These findings hinted that increasing physical effort associated with reminder-setting made people more reluctant to offload and led to poorer task performance. This effect was especially pronounced at higher memory loads.

Moreover, we anticipated that the option to set reminders, regardless of effort, would improve accuracy at all memory loads. Our results showed that reminders improved target accuracies at memory loads 4 and 6, but the same could not be observed at load 2 (see Table 2). Participants achieved near-perfect accuracies at this load even when reminders were not allowed (*M* = .96, *SD* = .10), meaning that they could easily remember the two special circles without a need for external reminders. However, as the memory load increased, using reminders did improve task performance. This finding is consistent with past literature showing the benefits of reminders or other external tools in augmenting our memory (Gilbert et al., 2020; Storm & Stone, 2015).

Participants generally set more reminders as memory load increased (see Table 3, Figure 3), which was in line with our hypothesis as well as previous studies (Gilbert, 2015a; Scarampi & Gilbert, 2021). This was evidence that participants were strategic in their use of external aid to compensate for increases in task difficulty. However, notably at the high-effort condition, a significant increase in externalising proportion was only observed from loads 4 to 6, but not from loads 2 to 4 (see Table 3). In other words, higher-effort reminders compromised the compensatory offloading of intentions at low memory loads, and participants were more likely to change their offloading strategy, that is, set more reminders, when they faced six special circles. This finding suggests that people might be more resistant to increased offloading in response to task difficulty when reminders demand high effort.

Our main line of investigation concerns how increasing physical effort influences intention offloading. As predicted, high-effort reminders significantly reduced offloading at all memory loads. The differences in externalising proportion between low- and high-effort conditions were large (see effect sizes in Table 4). This supports our suggestion that cognitive offloading can be seen as a form of cost/benefit or value-based decision-making (Gilbert, 2023; Gilbert et al., 2020).

In terms of metacognition, this experiment replicated the results of past studies (Boldt & Gilbert, 2019; Engeler & Gilbert, 2020; Gilbert, 2015b; Gilbert et al., 2020; Grinschgl et al., 2021), demonstrating underconfidence in participants’ unaided memory at all three memory loads (see Table 5, Figure 4). The extent of underconfidence, or metacognitive bias, also increased as memory load increased. This finding agrees with the existing literature showing that underconfidence may be more pronounced in more difficult tasks (Cherkaoui & Gilbert, 2017; Gilbert, 2015b). However, the predicted negative correlation between confidence and reminder-setting was not obtained, at least when averaged over memory loads and effort conditions. It is unclear whether this reflects a difference between the present paradigm and previous ones (Boldt & Gilbert, 2019; Gilbert, 2015b; Gilbert et al., 2020) or simply a lack of power, considering that only 52 participants took part in this study.

Finally, we were interested in how physical effort influences people’s *selectivity* in intention offloading. We predicted that more effortful reminders would have a larger effect in reducing offloading at low memory loads, when the need for reminders is least. We demonstrated a significant interaction between memory load and effort condition in our externalising proportion data but surprisingly found that participants had greater reductions in offloading when there were six targets compared with when there were two (see Table 4), opposite to our prediction.

A possible explanation for this result is that our manipulation of effort was confounded with our manipulation of memory load. As the additional clicking task was implemented every time a reminder was set, the effort required at low memory loads was smaller (two targets: 10 clicks) than that at high memory loads (six targets: 30 clicks). Any interaction in our externalising proportion data could be a result of differences in physical effort between memory loads rather than strategic changes in offloading behaviour (selectivity). Hence, we cannot conclusively interpret this interaction as an indicator of effort-induced selectivity. The purpose of Experiment 2 was to remove this confound and investigate whether a fixed difference in physical effort affects the selectivity in intention offloading.

## Experiment 2

Experiment 2 aimed to evaluate whether the impact of physical effort differed between memory loads when the manipulation required the same number of clicks at each load, rather than scaling with the total number of targets. This experiment repeated the procedures of Experiment 1 with one key change: the effort manipulation was adjusted by fixing the clicking task in the high-effort condition so that it required 15 clicks, but only for the *first* reminder set on each trial. This meant that the additional effort was constant at each memory load. We predicted that people would more selectively reduce offloading at low memory loads, that is, two-target condition. By contrast, there would be less reduction at high memory loads, that is, six-target condition, because of a stronger need for reminders.

### Methods

All experimental hypotheses, designs, procedures, and analyses were pre-registered at (https://osf.io/qky7j/) before data collection. Any deviations from this plan are clearly labelled.

#### Participants

Fifty-two participants were recruited online via Prolific (https://www.prolific.co) for consistency with Experiment 1. The earlier experiment found a significant interaction (*p* = .001) in externalising proportion between memory load and effort condition with a partial eta squared of .13. A sample size of 52 participants would give >99% power to detect an effect of the same size. Three participants whose mean target accuracy, averaged across all memory load and offloading conditions, exceeded 2.5 Median Absolute Deviation units were excluded and replaced with another three participants. The final sample included 52 participants (27 male, 25 female; mean age: 29 years; range: 19–57 years).

#### Procedures and data analysis

All procedures, analyses, and exclusion criteria were identical to Experiment 1, with the exception that the clicking task in the high-effort condition only appeared upon the first time a reminder was set on that trial, and it required the participant to click and close a window 15 times before they could proceed to the next circle. To view the experiment, visit http://samgilbert.net/demos/GC2. The code that runs the entire experiment can be found by visiting https://github.com/MetaOffloading/GC2.

### Results

Results of target accuracy and metacognitive judgements in this experiment were broadly similar to those in Experiment 1, and they will be displayed first. The main comparison in externalising proportion between Experiments 1 and 2 will follow.

#### Target accuracy

**Figure 5.**
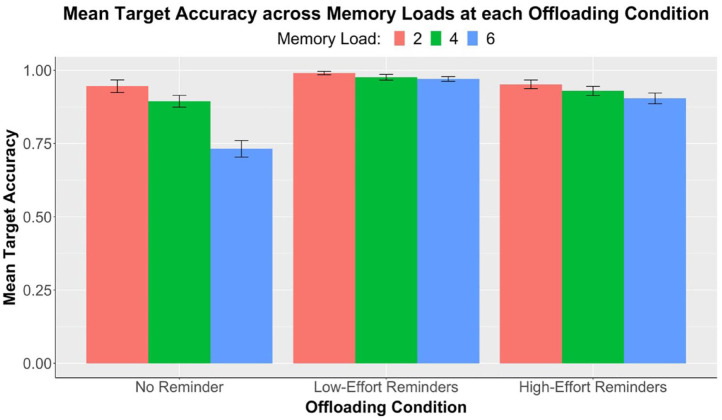
Experiment 2: Results of mean target accuracies (*n* = 52) across three levels of memory load, separately at each offloading condition. Error bars represent standard errors corrected for the within-subjects design using the method of Morey (2008).

See Figure 5 for a summary of the results. As in Experiment 1, a 3 × 3 repeated measures ANOVA with two factors of memory load (three levels) and offloading condition (three levels) revealed a significant main effect of memory load on target accuracy, *F*(1.84, 93.71) = 38.70, *p* < .001, η_p_^2^ = .43. Follow-up pairwise comparisons (Table 6) showed mostly significant results in the no-reminder condition, which was in line with findings in Experiment 1 showing that participants’ unaided task performance decreased as memory load increased. Whereas Experiment 1 showed a decrease in accuracy at higher memory loads in the high-effort but not low-effort condition, Experiment 2 showed a decrease in both conditions but only in the comparison between two- and six-target trials.

**Table 6.** Experiment 2: One-way ANOVAs (*F*-values, significance levels, and partial eta-square) and pairwise comparisons (*t*-values, effect sizes, and significance levels) of mean target accuracy between memory loads at each offloading condition. Refer to Figure 5 for mean differences.

	Offloading condition					
	No reminder		Low-effort reminders		High-effort reminders	
One-way within-subjects ANOVA	*F*(1.88, 96.10) = 33.53***, η_p_^2^ = .40		*F*(1.97, 100.56) = 2.33, η_p_^2^ = .04		*F*(1.82, 93.01) = 2.73^†^, η_p_^2^ = .05	
Pairwise comparisons between memory loads	*t*(51)	*d_z_*	*t*(51)	*d_z_*	*t*(51)	*d_z_*
Memory Loads						
2 vs. 4	1.83^†^	0.25	1.42	.20	1.10	.15
4 vs. 6	6.82***	0.95	0.61	.08	1.45	.20
2 vs. 6	7.21***	1.00	2.22*	.31	2.06*	.29

ANOVA: Analysis of variance.

† *p* < .1. *p < .05. **p < .01. ***p < .001.

A significant main effect of offloading condition on target accuracy was also found, *F*(1.65, 84.26) = 42.93, *p* < .001, η_p_^2^ = .46. Follow-up analyses demonstrated significant differences in accuracy at all loads between no-reminder and low-effort conditions, as well as between low-effort and high-effort conditions, whereas the comparison between no-reminder and high-effort conditions was only significant at load 6 (Table 7). Participants performed best when using low-effort reminders, compared with when using high-effort reminders or when reminders were not available. The interaction between memory load and offloading condition in our 3 × 3 ANOVA was also significant, *F*(3.01, 153.31) = 13.74, *p* < .001, η_p_^2^ = .21. For each pairwise comparison between two of the three offloading conditions, interactions were significant when the no-reminder condition was involved, no-reminder and low-effort: *F*(1.93, 98.32) = 23.48, *p* < .001, η_p_^2^ = .32; no-reminder and high-effort: *F*(1.81, 92.10) = 12.46, *p* < .001, η_p_^2^ = .20, suggesting that the effect of memory load on accuracy was the greatest in the no-reminder condition. Interaction was not significant in the comparison involving low-effort and high-effort conditions, *F*(1.76, 89.79) = .75, *p* = .46, η_p_^2^ = .01.

**Table 7.** Experiment 2: Pairwise comparisons (*t*-values, effect sizes, and significance levels) of mean target accuracy between offloading conditions at each memory load. Refer to Figure 5 for mean differences.

Pairwise comparisons between offloading conditions	Memory load					
	2		4		6	
	*t*(51)	*d_z_*	*t*(51)	*d_z_*	*t*(51)	*d_z_*
Offloading conditions						
No reminder vs. low-effort reminders	2.08*	.29	4.03***	.56	9.28***	1.29
No reminder vs. high-effort reminders	0.29	.04	1.42	.20	6.13***	.85
Low-effort reminders vs. high-effort reminders	2.47*	.34	2.98**	.41	3.57***	.49

† *p* < .1. *p < .05. **p < .01. ***p < .001.

#### Metacognitive evaluation

**Figure 6.**
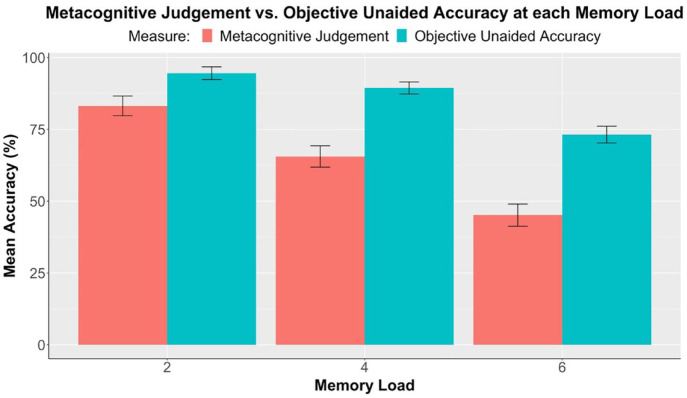
Experiment 2: Comparison between metacognitive judgements and objective unaided accuracy (*n* = 52), separately at each memory load. Error bars represent standard errors corrected for the within-subjects design using the method of Morey (2008).

As in Experiment 1, we calculated the metacognitive biases of each participant from their metacognitive judgements and unaided accuracies at each memory load. Figure 6 illustrates the comparison between the two variables.

A 2 × 3 repeated measures ANOVA with factors of memory load (three levels) and measure (two levels) demonstrated significant main effects from both factors, Memory load: *F*(1.70, 86.85) = 120.98, *p* < .001, η_p_^2^ = .70; Measure: *F*(1, 51) = 51.48, *p* < .001, η_p_^2^ = .50. Follow-up analyses further showed significant differences between the two measures at all loads, which indicated participants’ underconfidence in their unaided memory abilities (Table 8, Figure 6). We also found a significant interaction between memory load and measure, *F*(1.66, 84.45) = 10.23, *p* < .001, η_p_^2^ = .17, as the extent of underconfidence increased as memory load increased. These findings replicated the results of Experiment 1.

**Table 8.** Experiment 2: Comparison between metacognitive judgements and objective unaided accuracy at each memory load (*t*-values, effect sizes, and significance levels). Refer to Figure 6 for mean differences.

Paired samples *t-*tests comparing metacognitive judgements vs. objective unaided accuracy at each load		
	*t*(51)	*d_z_*
Memory loads		
2	4.09***	.57
4	6.08***	.84
6	6.71***	.93

† *p* < .1. *p < .05. **p < .01. ***p < .001.

Significant negative correlations between metacognitive evaluations and externalising proportion, averaged across all memory load and effort conditions, were found in this experiment, metacognitive judgement: *r*(50) = −.47, *p* < .001; metacognitive bias: *r*(50) = −.34, *p* = .01. Follow-up correlation analyses at each memory load also showed mostly significant results (see Tables 4 and 5 in the online Supplementary Material for details of all correlations). These results were contrasted with findings in Experiment 1, which provided inconclusive evidence regarding the relationship between confidence and offloading behaviour. Here, data in Experiment 2 supported our original hypothesis: participants who were less confident in their memory abilities tended to use more reminders.

#### Other analyses

Similar to Experiment 1, this experiment did not find consistently significant correlations between participants’ unaided accuracy and externalising proportion (see Table 6 in the online Supplementary Material for details of all correlations). Averaged across all memory loads and effort conditions, there was only a marginally significant effect.

#### Main comparison: externalising proportion

**Figure 7.**
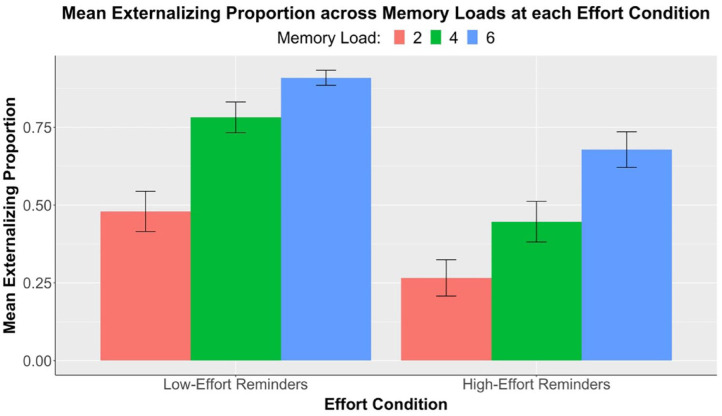
Experiment 2: Results of mean externalising proportions (*n* = 52) across three levels of memory load, separately at each effort condition. Error bars represent standard errors corrected for the within-subjects design using the method of Morey (2008).

With effort now fixed across all memory loads, we wanted to investigate how this modification changes people’s strategic decisions in using high-effort reminders. The same analyses as in Experiment 1 were run on the externalising proportion data. A 2 × 3 repeated measures ANOVA with factors memory load (three levels) and effort condition (two levels) demonstrated a significant main effect of memory load on externalising proportion, *F*(1.69, 86.28) = 50.75, *p* < .001, η_p_^2^ = .50. Pairwise comparisons further gave significant differences across all memory loads at each effort condition (see Table 9, Figure 7). We thus replicated findings from Experiment 1, suggesting that participants consistently set more reminders as memory load increased.

**Table 9.** Experiment 2: Pairwise comparisons (*t*-values, effect sizes, and significance levels) of mean externalising proportion between memory loads at each effort condition. Refer to Figure 7 for mean differences.

Pairwise comparisons between memory loads	Effort condition			
	Low-effort reminders		High-effort reminders	
	*t*(51)	*d_z_*	*t*(51)	*d_z_*
Memory loads				
2 vs. 4	5.73***	.79	3.96***	.55
4 vs. 6	3.12**	.43	4.32***	.60
2 vs. 6	6.99***	.97	7.17***	.99

† *p* < .1. *p < .05. **p < .01. ***p < .001.

We also replicated the significant main effect of effort condition on externalising proportion, *F*(1, 51) = 38.34, *p* < .001, η_p_^2^ = .43, with follow-up analyses confirming significant differences between low- and high-effort conditions at each load (Table 10). Once again, participants offloaded significantly less when reminders became more effortful at all memory loads. However, unlike Experiment 1, we did not find a significant interaction between memory load and effort condition in this experiment, *F*(1.91, 97.52) = 2.19, *p* = .12, η_p_^2^ = .04. Therefore, the interaction did not support our prediction that higher-effort reminders should selectively decrease offloading at lower memory loads.

**Table 10.** Experiment 2: Comparison of externalising proportion between effort conditions at each memory load (mean differences, *t*-values, effect sizes, and significance levels).

Paired samples *t-*tests comparing low-effort vs. high-effort reminders at each load			
	Mean difference	*t*(51)	*d_z_*
Memory loads			
2	.21	4.00***	.55
4	.34	5.48***	.76
6	.23	4.48***	.62

† *p* < .1. *p < .05. **p < .01. ***p < .001.

To further investigate this unexpected result, we looked into the mean differences in externalising proportion between low- and high-effort conditions at each load, and compared the differences with those in Experiment 1. This investigation was not included in our pre-registered plan. The comparison is displayed in Table 11.

**Table 11.** Comparison of mean externalising proportion between Experiments 1 and 2 at each effort condition.

Effort condition	Memory load	Mean externalising proportion	
		Experiment 1	Experiment 2
Low-effort reminders	2	.41	.48
	4	.69	.78
	6	.84	.91
High-effort reminders	2	.12	.27
	4	.19	.45
	6	.38	.68
Difference between low- and high-effort conditions (low–high)	2	.29	.21
	4	.50	.34
	6	.46	.23

A different pattern can be observed from the two experiments. In Experiment 1, the reduction in offloading was larger at loads 4 and 6 (.50 and .46, respectively) and smallest (.29) at load 2. In Experiment 2, where physical effort associated with reminder-setting was fixed, reduction in offloading was largest at load 4 (.34), whereas loads 2 and 6 had similarly small reductions of .21 and .23. To evaluate this statistically, we ran a polynomial contrast for the differences in Experiment 2 and confirmed that the mean differences in externalising proportion across three loads followed a significant quadratic effect but no linear effect, linear: *t*(102) = .27, *p* = .79; quadratic: *t*(102) = 2.08, *p* = .04. In Experiment 1, this was not the case, as the same analysis revealed a highly-significant linear trend, as well as a quadratic one, linear: *t*(102) = 2.99, *p* = .004; quadratic: *t*(102) = 2.44, *p* = .02.

### Discussion

Target accuracy results were similar to those in Experiment 1. When reminders were not allowed, participants’ performance in the task decreased as the memory load increased. The drop in accuracy was effectively compensated by the use of low-effort reminders. Interestingly, in this experiment, accuracies in the high-effort condition did not show significant differences across memory loads despite still demonstrating a negative trend (Figure 5). This was not the case in Experiment 1, and the contrast could potentially be attributed to how effort was manipulated differently in the two experiments. When effort was unfixed (up to 30 clicks in Experiment 1), participants may be easily discouraged by the high number of additional clicks at high memory loads. Consequently, this could translate into increasingly poor task performance as difficulty increased. However, when effort was fixed (15 clicks in Experiment 2), participants may be generally more willing to set reminders regardless of changes in memory load, which resulted in more consistent task performance in the high-effort condition.

In terms of metacognitive confidence, we also replicated findings from Experiment 1. Participants demonstrated underconfidence in their unaided memory abilities, and the extent of metacognitive bias increased with memory load, consistent with previous results (Cherkaoui & Gilbert, 2017; Gilbert, 2015b). Furthermore, we demonstrated a significant negative correlation between metacognitive evaluations and externalising proportion in this experiment, which was not observed in Experiment 1. This correlation provided evidence that individuals with lower confidence in their memory tend to employ more reminders when they are available, as found in previous studies (Boldt & Gilbert, 2019; Gilbert, 2015b; Gilbert et al., 2020).

Turning to externalising proportion, we established significant main effects of memory load and effort condition on offloading. This supports our initial hypotheses that people set more reminders when memory load increases and that offloading is generally reduced when reminders become more effortful. Participants were more willing to use high-effort reminders when the effort manipulation was fixed (Experiment 2) compared with when it was unfixed (Experiment 1). The mean differences between low- and high-effort conditions at all memory loads were also smaller in Experiment 2 than in Experiment 1 (Table 11). In other words, there was an overall smaller reduction in offloading when physical effort was fixed. This further supports the above interpretation that participants readily set more high-effort reminders when effort manipulation was not influenced by memory load.

The key investigation of this experiment concerns whether a fixed increase in physical effort induces selectivity in participants’ offloading behaviour in response to changing memory loads. Although we predicted that increasing physical effort would most strongly reduce offloading at the lowest memory load, the results of this experiment did not support this hypothesis. If anything, a higher effort most strongly reduced offloading behaviour at an intermediate memory load (i.e. load 4 in this experiment), but this result should be interpreted with caution because the relevant analysis was not pre-registered.

## General discussion

This study was the first to explore the role of physical effort associated with setting a reminder in strategic intention offloading. Past literature has revealed a number of cognitive and metacognitive factors that influence our cost-benefit analysis when making offloading decisions. The present results show that physical effort is another factor that should be taken into consideration.

Experiments 1 and 2 manipulated the physical effort of reminder-setting in two different ways: effort was unfixed and influenced by the number of special circles in Experiment 1, whereas Experiment 2 demanded a constant amount of additional effort when setting reminders. Both experiments showed that participants had the poorest task performance when they were not allowed to set reminders, and their performance decreased as memory load increased. The availability of low-effort reminders effectively compensated for the rising task difficulty, and participants were able to consistently achieve near-perfect accuracies with the aid of reminders. Other studies have also demonstrated how the provision of reminders improves prospective memory task performance (Henry et al., 2012; Jones et al., 2021; Maylor, 1990). Our finding in this study further supports the practical effectiveness of intention offloading.

Increasing physical effort, however, negatively affects this compensatory effect of offloading, which in turn also leads to declining task performance. There were significant reductions in offloading behaviour at all memory loads when reminders became more effortful, and the reductions were even greater when effort manipulation was unfixed. This effect of physical effort as a cost in offloading is comparable to the findings of another study conducted by Grinschgl et al. (2020). In their study, participants engaged in less offloading when using mouse-based (higher effort) compared with touch-based controls (lower effort), and when the reminder interface was less responsive (higher temporal cost). Whereas Grinschgl and colleagues (2020) investigated the nature of reminder-setting in a working memory task, our study demonstrated a similar effect of physical effort in the context of intention offloading.

We predicted that the effect of increased physical effort would be most evident at low memory loads because the stronger need for reminders at high loads would offset the cost of physical effort. To our surprise, Experiment 1 showed an opposite result, as participants had the smallest reduction in offloading at load 2. Experiment 2, which removed the confound between memory load and effort manipulation by fixing the number of additional clicks, also did not provide conclusive evidence for our predicted selectivity. Although Experiment 1 showed a strong linear trend in the mean differences in externalising proportion across the three loads, Experiment 2 did not. This might reflect the confound between memory load and the effort manipulation which was present in Experiment 1 but not Experiment 2. Furthermore, both experiments showed a significant quadratic effect with the greatest reduction in high-effort reminder-setting at the intermediate memory load. We tentatively suggest one possible explanation for this: participants may be more sensitive to the physical effort of reminder-setting when they are most unsure about which strategy to use. When the memory load was lowest or highest, participants may have been relatively confident in their strategy choice—with only two special circles, there was no need to set reminders, especially because they required extra time and effort, whereas at load 6, participants might always rely on reminders because of how difficult the task was. By contrast, they may have been less certain of the correct strategy at the intermediate memory load (load 4), making them more sensitive to other factors such as physical effort. This hypothesis could be tested in future experiments by asking participants to provide confidence ratings about their strategy choices.

The current study adds to the existing evidence showing that intention offloading is a delicate process of cost-benefit decision-making. Remembering an intention internally and setting (effortful) reminders externally incur respective costs and benefits. The costs of remembering involve an expense of cognitive effort and an increased likelihood to forget, but this has the benefit of saving the time/physical effort spent setting reminders. Offloading, on the contrary, takes more physical effort, but almost guarantees that the intention would be remembered and fulfilled. Deciding which type of effort to expend, therefore, likely depends on the context of the situation as well as task characteristics. One key factor governing this decision might be the subjective value of intentions. Existing literature has shown that people are more inclined to offload high-value intentions (Dupont et al., 2023). Hence, when an intention is subjectively important to an individual, the costs of forgetting might be too high such that they would resort to external tools even if extra physical effort is demanded. Furthermore, a second factor would be the length of time between creating the intention and its execution (Conte & McBride, 2018; McBride et al., 2013). A person would more likely set a reminder for a meeting 2 weeks later, compared with one that takes place tomorrow. Longer delay periods between intention formation and fulfilment mean that more cognitive effort is required to hold the intention, which again raises the chance of forgetting. In this case, the preference to avoid cognitive effort outweighs the physical effort of setting a reminder, and it would be more reasonable to store the intention externally. In sum, these factors are interesting lines of research that warrant separate studies to investigate how physical effort plays into individual cost-benefit calculations of offloading.

Finally, understanding the influence of physical effort on intention offloading has direct real-world implications. In the age of technological and digital boom, our cognitive systems are becoming ever more integrated with external tools. These tools demand different levels or types of effort depending on their design and interfaces. Unlike an experimental task, we often have greater flexibility in devising strategies to offload in our everyday lives, and the amount of effort for setting a reminder can be adjusted personally. Understanding the factors that influence decisions whether or not to use those tools and designing the tools in a way that optimises such decisions, can play a positive role in facilitating the adaptive use of cognitive technology.

## Supplemental Material

sj-docx-1-qjp-10.1177_17470218231199977 – Supplemental material for Influence of the physical effort of reminder-setting on strategic offloading of delayed intentions Supplemental material, sj-docx-1-qjp-10.1177_17470218231199977 for Influence of the physical effort of reminder-setting on strategic offloading of delayed intentions by Gavin Chiu and Sam J Gilbert in Quarterly Journal of Experimental Psychology
