# Supplementary material for: Influence of the physical effort of reminder-setting on strategic offloading of delayed intentions
Source: Q J Exp Psychol (Hove). 2023 Sep 23;77(6):1295–311. doi: 10.1177/17470218231199977 (PMC11103908; doi:10.1177/17470218231199977)
Supplement: sj-docx-1-qjp-10.1177_17470218231199977 – Supplemental material for Influence of the physical effort of reminder-setting on strategic offloading of delayed intentions [file sj-docx-1-qjp-10.1177_17470218231199977.docx]

**Influence of the physical effort of reminder-setting on strategic offloading of delayed intentions**

Gavin Chiu & Sam J. Gilbert

Institute of Cognitive Neuroscience, University College London, UK

**Author note**

Data and code to reproduce all analyses, along with the original study pre-registrations can be found at https://osf.io/qky7j/

**Supplementary Information**

***Supplementary Table 1***

*Experiment 1: Correlations between metacognitive judgement and externalizing proportion under different conditions.*

| **Correlations between metacognitive judgement and externalizing proportion** | | ***r*(50)** | ***p*** |
| --- | --- | --- | --- |
| **Conditions** | Averaged across all memory load and effort conditions | -.16 | .24 |
|  | Load 2, averaged across effort conditions | -.30 | .03* |
|  | Load 4, averaged across effort conditions | -.23 | .11 |
|  | Load 6, averaged across effort conditions | -.09 | .52 |

*Note: ^#^p < .1. *p < .05. **p < .01. ***p < .001.*

***Supplementary Table 2***

*Experiment 1: Correlations between metacognitive bias and externalizing proportion under different conditions.*

| **Correlations between metacognitive bias and externalizing proportion** | | ***r*(50)** | ***p*** |
| --- | --- | --- | --- |
| **Conditions** | Averaged across all memory load and effort conditions | -.09 | .52 |
|  | Load 2, averaged across effort conditions | -.19 | .17 |
|  | Load 4, averaged across effort conditions | -.05 | .73 |
|  | Load 6, averaged across effort conditions | .04 | .79 |

*Note: ^#^p < .1. *p < .05. **p < .01. ***p < .001.*

***Suppementary Table 3***

*Experiment 1: Correlations between objective unaided accuracy and externalizing proportion under different conditions.*

| **Correlations between objective unaided accuracy and externalizing proportion** | | ***r*(50)** | ***p*** |
| --- | --- | --- | --- |
| **Conditions** | Averaged across all memory load and effort conditions | -.34 | .01* |
|  | Load 2, averaged across effort conditions | -.17 | .23 |
|  | Load 4, averaged across effort conditions | -.22 | .12 |
|  | Load 6, averaged across effort conditions | -.19 | .18 |
|  | Load 2, Low-effort reminders | -.21 | .14 |
|  | Load 2, High-effort reminders | -.05 | .75 |
|  | Load 4, Low-effort reminders | -.15 | .28 |
|  | Load 4, High-effort reminders | -.23 | .11 |
|  | Load 6, Low-effort reminders | -.29 | .04* |
|  | Load 6, High-effort reminders | -.05 | .73 |

*Note: ^#^p < .1. *p < .05. **p < .01. ***p < .001.*

***Supplementary Table 4***

*Experiment 2: Correlations between metacognitive judgement and externalizing proportion under different conditions.*

| **Correlations between metacognitive judgement and externalizing proportion** | | ***r*(50)** | ***p*** |
| --- | --- | --- | --- |
| **Conditions** | Averaged across all memory load and effort conditions | -.47*** | < .001 |
|  | Load 2, averaged across effort conditions | -.44** | .001 |
|  | Load 4, averaged across effort conditions | -.44** | .001 |
|  | Load 6, averaged across effort conditions | -.34* | .01 |

Note: ^#^*p* < .1. **p* < .05. ***p* < .01. ****p* < .001.

***Supplementary Table 5***

*Experiment 2: Correlations between metacognitive bias and externalizing proportion under different conditions.*

| **Correlations between metacognitive bias and externalizing proportion** | | ***r*(50)** | ***p*** |
| --- | --- | --- | --- |
| **Conditions** | Averaged across all memory load and effort conditions | -.34* | .01 |
|  | Load 2, averaged across effort conditions | -.44** | .001 |
|  | Load 4, averaged across effort conditions | -.23 | .11 |
|  | Load 6, averaged across effort conditions | -.31* | .02 |

Note: ^#^*p* < .1. **p* < .05. ***p* < .01. ****p* < .001.

***Supplementary Table 6***

*Experiment 2: Correlations between objective unaided accuracy and externalizing proportion under different conditions.*

| **Correlations between objective unaided accuracy and externalizing proportion** | | ***r*(50)** | ***p*** |
| --- | --- | --- | --- |
| **Conditions** | Averaged across all memory load and effort conditions | -.25^#^ | .07 |
|  | Load 2, averaged across effort conditions | -.07 | .61 |
|  | Load 4, averaged across effort conditions | -.31* | .03 |
|  | Load 6, averaged across effort conditions | .03 | .81 |
|  | Load 2, Low-effort reminders | -.02 | .87 |
|  | Load 2, High-effort reminders | -.11 | .44 |
|  | Load 4, Low-effort reminders | -.13 | .34 |
|  | Load 4, High-effort reminders | -.34* | .01 |
|  | Load 6, Low-effort reminders | .12 | .39 |
|  | Load 6, High-effort reminders | -.01 | .93 |

Note: ^#^*p* < .1. **p* < .05. ***p* < .01. ****p* < .001.
